# Identifying the diagnostic gap of tardive dyskinesia: an analysis of semi-structured electronic health record data

**DOI:** 10.1186/s12888-025-06780-w

**Published:** 2025-04-21

**Authors:** Kira Griffiths, Yida Won, Zachery Lee, Lu Wang, Christoph U. Correll, Rashmi Patel

**Affiliations:** 1 Holmusk Technologies Inc., UK, 414 Linen Hall, 162-168 Regent Street, London, W1B 5 TE UK; 2 Holmusk Technologies Inc., Singapore, Blk 71, Ayer Rajah Crescent, Singapore, Singapore; 3 Zucker Hillside Hospital, Department of Psychiatry, Northwell Health, Glen Oaks, NY USA; 4 Donald and Barbara Zucker School of Medicine at Hofstra/Northwell, Department of Psychiatry and Molecular Medicine, Hempstead, NY USA; 5 The Feinstein Institute for Medical Research, Center for Psychiatric Neuroscience, Northwell Health, New Hyde Park, NY USA; 6 Department of Child and Adolescent Psychiatry, Charité - Universitätsmedizin Berlin, Berlin, Germany; 7 German Center for Mental Health (DZPG), Partner Site Berlin, Berlin, Germany; 8 Department of Psychiatry, University of Cambridge, Cambridge, UK

**Keywords:** Tardive dyskinesia, Real-world data, Underdiagnosis, ICD, Electronic health record

## Abstract

**Background:**

Tardive dyskinesia (TD) is a severe and persistent involuntary movement disorder associated with long-term antipsychotic treatment. TD is likely underreported and misdiagnosed in routine practice, and there is a need to understand the proportion of patients who may experience TD but receive no formal diagnosis. This information could support the characterisation of patient populations that may benefit from novel therapeutic interventions. This study aimed to identify and describe patients with diagnosed or undiagnosed TD. Demographic and clinical features associated with an ICD-9/10 diagnosis of TD were explored.

**Methods:**

A retrospective study was conducted using de-identified electronic health record (EHR) data captured between 1999 and 2021 in the US. A cohort of 32,558 adults with schizophrenia-spectrum disorders, major depressive disorder with psychosis or bipolar disorder with psychosis who were prescribed antipsychotics was selected. Abnormal movements associated with TD and presence of TD documented in semi-structured EHR data were extracted through manual review of text recorded as part of the mental state examination. Patients with a recorded diagnosis of TD were identified based on the presence ICD-9/10 codes within structured portions of medical records: ICD-9: 333.85; ICD-10: G24.01. Logistic regression was used to assess the association between patient characteristics and an ICD diagnosis.

**Results:**

Altogether, 1,301 (4.0%) patients had either description of abnormal movements associated with TD (*n*=691) or documented TD (*n*=610) within semi-structured EHR data. Of those patients, only 64 (4.9%) had an ICD-TD diagnosis in structured EHR data. When the cohort was limited to those with documented TD in semi-structured EHR data, 56 (9.2%) had an ICD-TD diagnosis. Black/African-American race was associated with lower odds of ICD diagnosis compared with white race (OR=0.46, 95%CI=0.20–0.95, *p*=0.04). Treatment in community mental health centres was associated with increased odds of an ICD diagnosis compared to an academic medical centre (OR=adjusted OR=2.02, 95%CI=1.09–3.74, *p*=0.03).

**Conclusions:**

This study highlights a pressing need for clinicians to better recognise and diagnose TD, which in turn may contribute to increased access to treatments for patients. A recorded ICD diagnosis of TD may be driven by factors related to both the patient and clinical setting.

**Supplementary Information:**

The online version contains supplementary material available at 10.1186/s12888-025-06780-w.

## Introduction

Tardive dyskinesia (TD) is a severe and persistent involuntary movement disorder characterised by persistent and repetitive abnormal movements of the face, lips, tongue, trunk and extremities [8, 32]. TD is primarily caused by long-term use of medications that block dopamine receptors and is therefore a side effect most frequently associated with antipsychotic treatment [5, 8, 12]. Patients with TD experience functional disability and reduced quality of life, high medical morbidity, and are at increased risk of poor antipsychotic medication adherence [6, 7, 19, 28]. Knowledge of risk factors [26] plus the timely diagnosis and treatment of TD are therefore a clinical priority, to reduce illness burden associated with long-term antipsychotic treatment and to promote treatment adherence to maximise antipsychotic therapeutic potential.

The estimated prevalence of TD in antipsychotic-treated populations ranges between 15–50% and varies according to clinical features of the cohort studied as well as study methodology [4, 16, 31, 32]. A meta-analysis of 41 studies found an overall mean prevalence of 25.3% in cohorts treated with antipsychotics [4], with evidence of increased risk of TD in patients treated with first-generation compounds [4, 5]. All studies in that review used a clinical rating scale to assess TD, with the Abnormal Involuntary Movement Scale most frequently adopted (AIMS; *n*=36 studies, 87.8%) [17]. TD prevalence estimates are lower in studies which use retrospective real-world clinical data ([Leo et al.; 20]). In part, this difference may be driven by the lack of formal assessment, recognition and documentation of movement disorders in at-risk populations in routine clinical care ([3, 14, 18, 22]). For example, one analysis of clinical data collected within a community mental health treatment centre found that half (51%) of patients treated with an antipsychotic had a clinical record of the AIMS [22], with TD being evident in less than 1% of patients. When TD was more broadly defined using billing codes or nursing notes, less than 1% of the overall study cohort had evidence of the disorder. Overall, evidence of underassessment and underreporting of TD in real-world clinical practice raises questions on the utility of real-world data sources for reliable identification and characterisation of patient populations affected by TD.

Vesicular monoamine transporter type-2 (VMAT-2) inhibitors are evidenced to successfully treat TD [27]. However, these pharmacological treatments may be underutilised in favour of other less-evidenced strategies, such as antipsychotic switching or dose reduction [2]. Given that there are FDA-approved and viable therapeutic options, an appropriate diagnosis of TD is important for timely treatment and to mitigate potential risks associated with less evidenced interventions [6]. A better description of the proportion of patients who experience abnormal movements associated with TD but who have no formal diagnosis could support the characterisation of cohorts that may benefit from novel therapeutic interventions, and thereby aid to close the treatment gap in TD.

Electronic health records (EHRs) can be used to describe large cohorts of patients who are representative of those receiving routine mental healthcare. EHRs typically include structured individual-level data on demographics, diagnoses, and prescribed medications. A further source of clinical information is recorded as free-text, which comprise clinician-documented notes that may describe clinical assessments and treatment plans. Here, the mental state examination (MSE) is a systematic and routine assessment used to describe the clinical presentation of a patient at the time of observation [21]. As part of the MSE, clinicians are to observe and assess the motor activity of an individual. These clinical observations may be recorded as free text within pre-set EHR fields, termed semi-structured data. Given that observation of motor behaviour in antipsychotic-treated populations is recommended at each clinical visit to identify possible TD [13], the MSE may hold potential to identify cohorts who experience TD. Complimentary EHR data may then promote characterisation of these cohorts in terms of routinely collected demographic and clinical information that may not be present in other real-world data sources, such as claims data.

This study analysed de-identified structured and semi-structured EHR data from speciality inpatient and outpatient mental healthcare centres to address three aims: (i) estimate the prevalence of diagnosed TD through recorded ICD code, (ii) estimate the prevalence of undiagnosed TD through analysis of semi-structured MSE data, and (iii) assess clinical features associated with diagnosed TD.

## Method

### Data source and ethical committee approval

This retrospective study used de-identified EHR data from the NeuroBlu database (release 22R1, June 2022; [24]). NeuroBlu 22R1 comprises longitudinal and patient-level clinical data captured between 1999 and 2021 across 25 U.S. healthcare centres. These centres provided secondary psychiatric care in both inpatient and outpatient settings, and patients were included into the NeuroBlu database if they were recorded with at least one mental health condition of any type. For the identified patients, all corresponding records available in the EHR system are collected. Retrospective EHR records have been standardised to the Observational Medical Outcomes Partnership Common Data Model (OMOP CDM) and made available in the NeuroBlu database, which is described in full in a published cohort profile ([24]). All data were de-identified at source using the Safe Harbor standards outlined in the HIPAA Privacy Law. Institutional Review Board (IRB) evaluation with a waiver of informed consent was obtained prior to study commencement (reference: WCG-IRB 1–1,470,336−1).

### Study population

This study included adult patients (≥ 18 years) with an ICD-9 or −10 diagnosis of a schizophrenia-spectrum disorder or an affective disorder with evidence of psychosis (Supplementary Tables 1 and 2). All patients had a record of any antipsychotic prescription within ± 1 years from the recorded psychiatric diagnosis. Antipsychotic prescriptions were required for a minimum duration of 14 days, to exclude cases where an antipsychotic may have been prescribed on a pro re nata basis rather than an ongoing treatment plan. Inclusion criteria also required an MSE record at any point within an individual’s EHR to facilitate identification of patients with clinician-recorded evidence of TD. If an individual had multiple MSE records, all were considered when identifying evidence of TD. Two sub-cohorts were identified: 1) patients with evidence of TD in semi-structured EHR data *without* an ICD-TD diagnosis in structured EHR data, and 2) patients with evidence of TD in semi-structured data *with* an ICD-TD diagnosis.

#### Identifying diagnosed TD in structured EHR data

Patients with a recorded diagnosis of TD were identified based on the presence of an ICD-9: 333.85 or ICD-10: G24.01 code within structured EHR data. These codes were considered as diagnostic confirmation of TD given their requirement for billing purposes and thereby treatment eligibility for TD.

#### Identifying evidence of TD in semi-structured EHR data

EHR data were from secondary psychiatric care settings, which refers to specialised mental health services (as opposed to psychiatric treatment in primary care settings) that provide diagnosis and treatment for individuals with psychiatric conditions. Routine assessments such as the mental state examination can be used in these care settings to monitor treatment effectiveness and tolerability. The mental state examination (MSE) was therefore used to identify evidence of TD in semi-structured EHR data. A total of 25,376 unique text-strings which described patient psychomotor function were independently reviewed for keywords by one researcher specialised in psychotic disorders and one consultant psychiatrist. Here, keywords were derived using the AIMS assessment tool, and text-strings which described abnormal movements associated with TD or which directly referenced presence of TD were identified (Supplementary Tables 3 and 4). Patients with evidence of TD were identified based on the presence of at least one text-string which described abnormal movement associated with TD or directly referenced presence of TD.

### Variables and outcome

The index event was defined as the start date of the first recorded antipsychotic prescription. The following demographic and clinical data were extracted at the index event: age, sex, race, care setting type. In addition, all psychiatric diagnoses recorded at the index event ± 1 year, and all prescribed antipsychotics recorded at the index event ± 90 days were extracted. Anticholinergic prescriptions were extracted (± 90 days of the index event), given that these compounds may be prescribed for the treatment of extrapyramidal side effects which may be difficult to distinguish from TD [9]. Given the possibility that the documented presence of abnormal movements due to motor tics or stereotypies may be related to disorders other than TD, the presence of comorbid diagnoses of autism-spectrum disorder and obsessive–compulsive disorder [15, 30] were also extracted. Operational definitions for all extracted variables are reported in Supplementary Table 5. For regression analyses, the outcome of interest was ICD-TD diagnosis (yes/no).

### Statistical analysis

The proportion of antipsychotic-exposed patients with evidence of abnormal movement and/or TD in semi-structured data was calculated, as well as the proportion of patients with an ICD-TD diagnosis, and their overlap. To better understand the specificity of semi-structured text-strings, the proportion of patients with documented presence of TD vs. descriptions of abnormal movements associated with TD was calculated. In cases where an individual had both description of abnormal movements associated with TD and TD directly documented, the individual was categorised as documented presence of TD.

Demographic and clinical characteristics of the population with abnormal movement and/or TD documented in semi-structured portions of the EHR were described. The population was then stratified based on presence or absence of an ICD-TD diagnosis, and the sub-cohorts were described using mean (SD) for continuous variables and number (%) for categorical variables. Characteristics of the sub-cohorts were compared to explore differences in the data distribution between the groups. Mann–Whitney U was used for continuous variables, while Chi-squared or Fisher’s exact test (for cells with < 5 observations) was used for categorical data.

Univariable logistic regression analysis was then used to explore associations between demographic and clinical characteristics and an ICD-TD diagnosis. *P*-values were Bonferroni-corrected to adjust for multiple comparisons, where a *p*-value < 0.006 (0.05/9) was considered statistically significant. Subsequently, all exposure variables were entered into one multivariable model to examine adjusted associations between demographic and clinical characteristics with presence of an ICD-TD code (yes/no). The independent variables of interest were age, sex, race, anticholinergic prescription, first-generation antipsychotic prescription, second-generation antipsychotic prescription, multiple antipsychotic prescriptions, care setting type, and specificity of semi-structured text-string (description of abnormal movements associated with TD vs. TD directly documented) (Supplementary Table 5). For categorical covariates, the category with the highest number of observations was used as the reference. In this adjusted model, a *p*-value < 0.05 was considered significant.

### Software

The Structured Query Language (SQL) program was used for data extraction from NeuroBlu and R version 4.2.0 was used for statistical analysis.

## Results

A total of 32,558 patients met study inclusion criteria. Of those patients, 31,256 (96.0%) had no evidence of abnormal movements associated with TD or documented presence of TD (mean ± SD age=42.6 ± 14.6 years, 50.0% female). Most patients were white (46.4%), followed by black or African-American (24.3%) and unknown race (22.7%). Cohort assembly also identified an additional 148 patients who had an ICD-TD diagnosis without evidence of TD recorded in semi-structured EHR data.

### Frequency of abnormal movements or presence of TD in semi-structured EHR data

Altogether, 1,301 (4.0%) patients had evidence of TD in the MSE (mean ± SD age=46.8 ± 14.1 years, 56.3% female). Of those patients, 64 (4.9%) had a documented ICD-TD diagnosis. Most patients were white (*n*=703, 53.9%), followed by black or African-American (*n*=299, 23.0%) and unknown race (*n*=242, 18.6%). Of those patients, 610 (46.9%) had documented presence of TD (e.g. “tardive dyskinesia”, “TD evident”) and 691 (53.1%) patients had descriptions of abnormal movements associated with TD (e.g. “mild oral buccal movements”; Supplementary Table 3). Of those with documented presence of TD, 56 (9.2%) had an ICD-TD diagnosis.

All text-strings categorised by body location are reported in Supplementary Table 4. The most frequently recorded text-strings described abnormal extremity movements (*n*=703, 54.0%) such as “wringing of hands” and “variable repetitive flexing of wrist”, followed by unspecified symptoms (*n*=482, 37.0%), such as “tardive dyskinesia present”. Abnormal truncal movements, such as “rocking back and forth” and “hip gyrations”, were documented in 127 (9.8%) patients. Abnormal facial or oral movements, such as “oral buccal smacking/puckering/chewing”, were documented in 104 (8.0%) patients. Most patients had abnormal movements documented in one location (*n*=1,214, 93.3%).

Demographic and clinical characteristics of patients with abnormal movement recorded in semi-structured portions of the EHR with vs. without an ICD-TD diagnosis are reported in Table 1.

**Table 1:** Demographic and clinical characteristics of patients with evidence of tardive dyskinesia in the mental state examination (total *n*=1,301), grouped by presence or absence of an ICD-TD diagnosis

Variable	Total (*n*=1,301)	ICD-TD code present (*n*=64)	ICD-TD code absent (*n*=1,237)	*p*-value
Age (mean ± SD, years)	46.8 ± 14.1	52.3 ± 12.6	46.5 ± 14.1	0.001
Sex (female)	733 (56.3)	34 (53.1)	699 (56.5)	0.69
Race				0.11
- White	703 (54.0)	43 (67.2)	660 (53.4)	
- Black or African American	299 (23.0)	10 (15.6)	289 (23.4)	
- Asian	12 (1.0)	< 5 (< 10.0)	5–10 (< 1.0)	
- Other	45 (3.5)	< 5 (< 10.0)	40–45 (< 5.0)	
- Unknown	242 (18.7)	7 (10.9)	235 (19.0)	
Anticholinergic prescription	274 (21.1)	20 (31.3)	254 (20.5)	0.06
First-generation antipsychotic prescription	319 (24.5)	22 (34.4)	297 (24.0)	0.08
Second-generation antipsychotic	1,093 (84.0)	46 (71.9)	1,047 (84.6)	0.01
Multiple antipsychotic prescriptions	234 (18.0)	8 (12.5)	226 (18.3)	0.31
Autism / Asperger’s Syndrome diagnosis	< 5 (< 1.0)	< 5 (< 1.0)	< 5 (< 1.0)	1.00
Obsessive–compulsive disorder diagnosis	40 (3.1)	< 5 (< 10.0)	35–40 (< 4.0)	0.72
Site type				0.09
- Academic medical centre	787 (60.5)	32 (50.0)	755 (61.0)	
- Community mental health centre	353 (27.1)	25 (39.1)	328 (26.5)	
- Private practice	161 (12.4)	7 (10.9)	154 (12.4)	
MSE text-string specificity				5.81 × 10^–11^
- Documented presence of TD	610 (46.9)	56 (87.5)	554 (44.8)	
- Documented abnormal movements	691 (53.1)	8 (12.5)	683 (55.2)	

Data are presented as N (%) unless otherwise specified. *P*-values reflect unadjusted between-group comparisons. Mann–Whitney U was used for continuous variables, while Chi-squared or Fisher’s exact test (for cells with < 5 observations) was used for categorical variables. In accordance with HIPAA guidelines, counts for small sub-groups (< 5) have been anonymised, and ranges are used to ensure participant identity is protected. Other race includes native Hawaiian or other Pacific Islander, American Indian or Alaska Native, multiracial. In cases where an individual had both documented presence of tardive dyskinesia and documented abnormal movements in semi-structured MSE data, they were categorised under “documented presence of tardive dyskinesia” to preserve mutual exclusivity of groups for regression modelling

*MSE* mental state examination, *TD *tardive dyskinesia

### Demographic and clinical features of individuals with an ICD-TD diagnosis

Output from regression analyses is reported in Table 2. The largest effect was for MSE text-string specificity, whereby patients with presence of TD documented were 9.7 times more likely to receive an ICD-TD diagnosis than patients with description of abnormal movements associated with TD (adjusted OR=9.72, 95% CI=4.64–23.10, *p* < 0.0000001). Treatment within a community mental health centre was also associated with increased odds of an ICD-TD diagnosis (adjusted OR=2.02, 95% CI=1.09–3.74, *p*=0.03, whereas black or African-American race was significantly associated with reduced odds of an ICD-TD diagnosis compared with white race (adjusted OR=0.46, 95% CI=0.20–0.95, *p*=0.04).

**Table 2:** Output from logistic regression analysis to investigate associations between demographic and clinical characteristics and presence of an ICD-TD diagnosis in the total cohort of patients with abnormal movements or presence of TD recorded in semi-structured portions of the EHR (*n*=1,301)

Variable	Univariable		Multivariable	
	**OR (95% CI)**	***p*-value**	**OR (95% CI)**	***p*-value**
Age (mean ± SD)	1.03 (1.01 − 1.05)	0.001	1.01 (0.99 – 1.03)	0.27
Female sex	0.87 (0.53–1.45)	0.59	0.79 (0.46–1.35)	0.39
Race				
- White (ref)	-	-	-	-
- Black	0.53 (0.25–1.03)	0.08	0.46 (0.20–0.95)	0.04
- Asian	1.40 (0.08–7.43)	0.75	1.43 (0.07–10.58)	0.76
- Other	1.10 (0.26–3.17)	0.88	1.32 (0.29–4.27)	0.67
- Unknown	0.46 (0.19–0.96)	0.06	0.48 (0.19–1.10)	0.10
First-generation antipsychotic prescription	1.65 (0.96–2.79)	0.06	1.05 (0.24–4.30)	0.94
Second-generation antipsychotic prescription	0.46 (0.27–0.84)	0.008	0.82 (0.17–3.70)	0.80
Multiple antipsychotics prescribed	0.63 (0.28–1.28)	0.24	0.44 (0.14 – 1.13)	0.12
Anticholinergic	1.76 (1.00–3.00)	0.04	1.20 (0.65–2.26)	0.55
MSE text-string specificity				
- Abnormal movement (ref)	-	-	-	-
- Presence of TD	8.63 (4.32–19.74)	< 0.0000001	9.72 (4.64–23.10)	< 0.0000001
Clinical setting				
- AMC (ref)	-	-	-	-
- CMHC	1.80 (1.04–3.08)	0.03	2.02 (1.09–3.74)	0.03
- PP	1.07 (0.43–2.34)	0.87	0.79 (0.30–1.89)	0.62

Outcome = ICD-TD diagnosis (yes, *n*=64; no, *n*=1,237)

Other race = American-Indian or Alaska Native, Native Hawaiian or Other Pacific Islander, multiracial

*MSE* mental state examination, *AMC* academic medical centre, *CMHC* community mental health centre, *PP* private practice

Given the large association between MSE text-string specificity and odds of an ICD-TD diagnosis, a sensitivity analysis was performed where the cohort was limited to those with presence of TD specified in the MSE (*n* = 610). Results remained the same such that an ICD-TD diagnosis was less likely in patients of black or African-American race (adjusted OR=0.45, 95% CI=0.19–0.97, *p*=0.05) and more likely in patients treated within a community mental healthcare centre (adjusted OR=2.39, 95% CI=1.24–4.61, *p*=0.01).

## Discussion

This study aimed to identify the proportion of patients with a history of antipsychotic treatment who had description of abnormal movements associated with TD or presence of TD documented in semi-structured EHR data. Clinical and demographic characteristics associated with presence of an ICD-TD diagnosis were also explored. The main finding was that less than 5% of patients with evidence of abnormal movements indicative of TD or specific mention of TD recorded during mental state examination also had an ICD-TD diagnosis. When the population was restricted to those with a specific mention of TD during mental state examination, evidence of underdiagnosis remained in that ICD-TD diagnosis was recorded in less than 10% of patients. Lack of a TD diagnosis in 95% of patients with evidence of abnormal movements associated with TD could represent a substantial missed opportunity for appropriate diagnosis and related evidence-based treatment.

Approximately 4% of the antipsychotic-treated population had abnormal movements indicative of TD documented in semi-structured EHR data. The frequency of TD decreased to < 2% of the population when only those with direct mention of TD in semi-structured data were considered. This result is broadly in line previous studies which have used routinely collected clinical data to estimate the prevalence of TD. One study of U.S. electronic health record data from a community health centre reported < 1% of antipsychotic-treated patients with AIMS assessment had evidence of TD [22], and a second study of U.S. electronic health record data from a network of ambulatory, inpatient and emergency department sites reported TD annual prevalence of 1–2% [20]. The low proportion of patients with documented evidence of TD in real-world data studies compared to clinical research may in part be driven by lack of training, time and adequate resource to systematically assess TD across varied real-world clinical settings [6].

A total of 1,301 patients had evidence of abnormal movements documented in semi-structured EHR data, and 4.9% of these patients also had an ICD-TD diagnosis. While involuntary facial and oral movements may account for up to 60–80% of TD cases [11, 13], current results suggest that broader features of a “tardive syndrome” may be frequently documented during clinical assessment. A key limitation is the lack of specificity of semi-structured text-strings. Text-strings categorised in the current study as abnormal movements may reflect documentation of other movement-related disorders or motor symptoms, such as akathisia, Parkinsonism, tremor, or tic disorder. While there is some evidence of stereotypies and complex movements such as hand wringing and hip gyrations in TD [1, 25], these movements are frequently documented in other psychiatric and neurodevelopment disorders such as autism or OCD. However, over 96% of the cohort had no recorded diagnosis of these disorders. Further, it is possible that non-specific descriptors of abnormal movements may reflect self-soothing behaviours seen in anxiety disorders or are related to hallucinations or delusions inherent to psychosis-spectrum disorders in and of themselves. Nonetheless, when we removed patients with non-specific descriptions of abnormal movements to leave only those with TD directly specified, over 90% did not have an ICD-TD diagnosis. Taken together, these results suggest that TD is likely underdiagnosed within the context of structured ICD-TD coding within EHRs. Future research may further investigate relationships between other psychiatric or neurodevelopmental comorbidities and TD diagnosis in antipsychotic-treated populations.

Results have implications for both clinical research and clinical care. Real-world data studies which use ICD codes to identify cohorts of interest with TD may not capture a complete and representative cohort, which in turn may limit generalisability of research cohorts centred on TD. Going forward, future research could utilise semi-structured EHR data to identify patients with TD in lieu of a structured diagnostic code. In terms of clinical implications, a lack of a recorded ICD-TD diagnosis in 95% of patients with likely evidence of TD recorded in semi-structured fields could represent a substantial missed opportunity for treatment. This discordance is relevant in the context of billing procedures, which require an ICD diagnosis for reimbursement purposes and also evidence-based treatments in the U.S healthcare system [29]. Given that our total study period includes some calendar years before the approval of novel therapeutics for treatment of TD, lack of ICD recording may reflect lack of recognition or lack of perceived necessity to offer a diagnosis, which may be associated with increased stigma and lack of effective treatment options.

Regression analyses found that black/African-American race was significantly associated with reduced odds of an ICD-TD diagnosis compared to white race. White race patients with documented abnormal movements were 54% more likely to have a concomitant ICD-TD diagnosis. Previous research has indicated that black or African-American individuals may be at increased risk of developing TD compared to other racial or ethnic groups [10, 27]. This increase in risk has been observed across inpatient and outpatient populations [23, 33], and may be driven by several factors including differential access to healthcare, quality of treatment, prescribing patterns in terms of type and dosage of antipsychotic, as well as differences in drug metabolism pathways [10, 11]. Despite black/African-American race comprising 23% of the current study cohort, these patients were at significantly reduced odds of receiving an ICD-TD diagnosis. While disparities in TD diagnosis across racial groups are evident in this analysis, it is important to note that reduced odds of TD-ICD diagnosis in those of black/African-American race reflects a statistical association rather than a confirmed inequity. Crucially, our analysis did not account for other features which may also vary by race, such as clinical presentation, insurance status or healthcare access. Future studies should aim to estimate the contribution of these factors to time timely diagnosis of TD, and how this in turn may relate to potential inequalities in standard of care.

The preliminary finding of disparities in TD-ICD diagnosis across racial groups is important in the context of associations between TD and increased risk of antipsychotic treatment discontinuation. Given that antipsychotic discontinuation may lead to exacerbation of treated psychosis, a lack of recognition and treatment of TD may result in exacerbation of underlying illness severity in already vulnerable patient groups. Regression analyses also identified community care setting as being associated with higher odds of documented ICD-TD diagnosis than academic settings. This finding may at first seem counterintuitive, as one would associate academic settings as those with higher quality of assessment and care than community care settings. While this might not be true when it comes to the assessment and diagnosis of TD, it is also possible that patients in community care settings are more likely severely and chronically mentally ill, or be treated with first-generation antipsychotics, putting them at higher risk for TD [4, 5, 26].

### Strengths and limitations

This study has several strengths, including a large and representative cohort of patients who received routine mental healthcare across speciality treatment centres. However, there are several limitations to consider. EHR data were not recorded for research purposes and there are several established risk factors for TD which were not available for inclusion in the analyses, such as duration and dosage of antipsychotic medication, early emergence of acute extrapyramidal symptoms, chronicity or duration of psychotic illness, distinction between primary diagnosis and comorbidities, diabetes diagnosis or HIV-positive status [23, 26, 31]. In addition, other neurodevelopmental disorders associated with abnormal movements and treated with antipsychotics such as ADHD were not included. A related point is that routinely collected clinical data may be subject to documentation biases or variability in diagnostic coding practices across providers. Future studies using other real-world databases are required to replicate current findings and explore their applicability to broader patient populations. Further, EHR data were collected as part usual of mental healthcare. As such, data on the presence of neurological comorbidities, which may also be associated with abnormal movements, were not available for analysis. Due to the source EHR data format we were unable to investigate temporal relationships between abnormal movements documented in the MSE and antipsychotic prescribing. Relatedly, there were no data on history of treatments prior to entry into the EHR, or alternative treatments received at centres which are not captured in NeuroBlu. It is also possible that other ICD codes for movement disorders, such as G25.9 (unspecified extrapyramidal and movement disorder) and G25.71 (akathisia), may capture patients with TD-like symptoms. However, at least some of these codes may represent acute rather than tardive syndromes. Future research could investigate these additional codes to assess their overlap with TD diagnoses and further explore the potential for under-capture of TD in EHRs. A further limitation is that we were unable to ascertain information on the severity of abnormal movements or TD features and the psychosocial impact of TD. This lack of information is important in the context of real-world data as there may be a threshold effect, such that abnormal movements with a pronounced impact on patient functioning may be more likely recorded during clinical assessment. Future studies should ideally investigate co-occurrence of free-text descriptors of clinical features of TD and other scale-based measures of the disorder, such as the AIMS scale.

## Conclusion

Results support previous research to show an underreporting of TD in real-world datasets and support need to better recognise and document symptoms, given availabilities of evidence-based and FDA-approved therapies for TD [20]. Further, results suggest that evidence of abnormal movements and presence of TD is more frequently reported in clinical notes than in structured diagnostic EHR data. Future real-world data studies should consider the utility of semi-structured EHR data to identify and characterise cohorts at risk for and with TD.

## Supplementary Information

Supplementary Material 1.

## Data Availability

The data that support the findings of this study have been originated by Holmusk Technologies, Inc. Requests for data sharing by license or by permission for the specific purpose of replicating results in this manuscript can be submitted to publications@holmusk.com.
