# Supplementary material for: Identifying the diagnostic gap of tardive dyskinesia: an analysis of semi-structured electronic health record data
Source: BMC Psychiatry. 2025 Apr 21;25:407. doi: 10.1186/s12888-025-06780-w (PMC12013043; doi:10.1186/s12888-025-06780-w)
Supplement: Supplementary file 1 — Supplementary Material 1. [file 12888_2025_6780_MOESM1_ESM.docx]

**SUPPLEMENTARY MATERIAL**

**Identifying the diagnostic gap of tardive dyskinesia: an analysis of semi-structured electronic health record data**

**S Table 1.** ICD-10 codes for schizophrenia spectrum disorders and affective disorders with psychotic features

| **Code** | **Description** |
| --- | --- |
| F20.* | Schizophrenia |
| F21 | Schizotypal disorder |
| F22.* | Delusional disorders |
| F23.* | Brief psychotic disorder |
| F24 | Shared psychotic disorder |
| F25.* | Schizoaffective disorders |
| F28 | Other psychotic disorder not due to a substance or known physiological condition |
| F29 | Unspecified psychosis not due to a substance or known physiological condition |
| F32.3 | Severe depressive episode with psychotic symptoms |
| F33.3 | Recurrent depressive disorder, current episode severe with psychotic symptoms |
| F30.2 | Mania with psychotic symptoms |
| F31.2 | Bipolar affective disorder, current episode manic with psychotic symptoms |
| F31.5 | Bipolar affective disorder, current episode, depression, severe with psychotic symptoms |
| F31.64 | Bipolar disorder, current episode mixed, severe, with psychotic symptoms |

Note: * = all child codes listed within a given parent category

**S Table 2.** ICD-9 codes for schizophrenia spectrum disorders and affective disorders with psychotic features

| **Code** | **Description** |
| --- | --- |
| 295.* | Schizophrenic disorders |
| 297.* | Delusional disorders |
| 298.* | Other nonorganic psychoses |
| 296.04 | Bipolar I disorder, single manic episode, severe, specified as with psychotic behaviour |
| [296.14](http://www.icd9data.com/2012/Volume1/290-319/295-299/296/296.14.htm) | Manic affective disorder, recurrent episode, severe, specified as with psychotic behaviour |
| [296.24](http://www.icd9data.com/2012/Volume1/290-319/295-299/296/296.24.htm) | Major depressive affective disorder, single episode, severe, specified as with psychotic behaviour |
| [296.34](http://www.icd9data.com/2012/Volume1/290-319/295-299/296/296.34.htm) | Major depressive affective disorder, recurrent episode, severe, specified as with psychotic behaviour |
| [296.44](http://www.icd9data.com/2012/Volume1/290-319/295-299/296/296.44.htm) | Bipolar I disorder, most recent episode (or current) manic, severe, specified as with psychotic behaviour |
| [296.54](http://www.icd9data.com/2012/Volume1/290-319/295-299/296/296.54.htm) | Bipolar I disorder, most recent episode (or current) depressed, severe, specified as with psychotic behaviour |
| [296.64](http://www.icd9data.com/2012/Volume1/290-319/295-299/296/296.64.htm) | Bipolar I disorder, most recent episode (or current) mixed, severe, specified as with psychotic behaviour |

Note: * = all child codes listed within a given parent category

**S Table 3.** All raw text-strings documented within the mental status examination portion of electronic records which may be indicative of tardive dyskinesia (TD), grouped by abnormal movements associated with TD vs. documented presence of TD

| **Documented presence of tardive dyskinesia** |
| --- |
| “Tardive Dyskinesia”, “TD”, “Tardive diskinesia (TD) present”, “evidence of tardive dyskinesia”, “mild TD”, “movements of tardive dyskinesia”, “TD evident”, “TD noted”, “possibly tardive dyskinesia”, “TD like movements that are min. worse from last we”, “Mild Tardive Dyskinesia”, “difficult to read because of tardive, but overall pleasant”, “EPS, and TD”, “marked tardive dyskinesia”, “mild tardive type movements”, “mild TD sx that have greatly improved”, “no abnormal movements aside from mild td-like”, “still tardive dyskinesia”, “td movements”, “TD, missing dentures”, “Tardive Dyskinesia of tongue“, “mouth with tardive syskinesia“,“Mild Tardive Dyskinesia of tongue“,“Severe TD orobuccal“,“tardive dyskinesia with orofacial movements“,“tardive dyskinesia with oralfacial movements“,“oral tardive dyskinesia“,“tongue movements consistent with TD“,“Slight tongue darting/Tardive dyskinesia“,"TD upper extremities, oral", “some TD of tongue“,“tardive dyskinesia with chronic involuntary mouth movements“,“?TD in the mouth“,“Facial grimacing/TD present consistent with previous exams.“, "Mild tardive dyskinesia, mostly of tongue", “some 'deepbreaths" suggestive of TD“,“Tardive dyskinesia of the tongue“,“TD of face“,“Possible TD in face; eyebrow movments and occasional lip smacking“,“TD symptoms affecting her mouth. No pillrolling tremor appreciated by me today.“, “orobuccal TD movements“,“questional tardive dyskinesia involuntary movements of lower jaw“,“Oral-buccal TD“,“Improved TD (abnormal lip movements on admission)“ , “restless; possible oral- lingual TD + akathisia“, “mild TD in her lips“, “mild tardive dyskinesia of the mouth“,“R hemifacial tic 2/2 TD“,"nervousness, orolingual movements (TD)", “Fine tremor and mild perioral TD“,“Oral TD“,"mild akasthisia, severe TD orofacial", “perioral movements c/w mild TD“,"TD of face, tongue, BUE", “Tardive dyskinesia (orofacial) “,“TD facial movements improved“,“mild oralbuccal TD“,"Clenched jaw, chattering movement, possible TD", “tardive dyskinesia with lip smacking“,“perioral TD“,“some buccooral dyskinesia suggestive of mild TD“,“involuntary mouth movement consistent with TD“,“Adventitious mouth movements ?TD“,“TD notable esp in jaw“,“probable oral TD“, “possible mouth TD“, “mouth/tongue TD“, “some lips smacking noted suggestive of TD“,“TD noted in mouth/tongue/jaw“, “repetitive tongue movements- reports h/o TD that is unchanged“, “possible tardive dyskinesia when speaking“,“oral movements: TD“,“ocular and jaw tardive dyskinesias“,“Clicking of mouth (possibly tardive dyskinesia)“, “TD observed in perioral muscles and tongue“, “Perioral tardive dyskinesia“, “Mild tardive dyskinesia“, “minimal oral TD movement“,“? repetitive oral movements; ?TD“,“Mild tardive dyskinesia of tongue“,“TD of tongue“,“Eyelid/Eyebrow twitches most likely TD“,“TD of face/mouth and B/L hand tremor“,"mouth movements, TD", “Oral buccal TD? “,"tardive dyskinesia , perioral movements.", “Oral Buccal TD“,"Tardive dyskinesia, continuous lip smacking", “mild oral-buccal TD movements seen on video“,“dyskinesia of mouth, trunk”, “mild-mod oral-buccal -lingual TD“, “Mild oral dyskinesia“, “Oral dyskinesia“,“Tardive Dyskinesia of mouth“,“chronic oral-bucal movments of td“,“TD of mouth“, “Tardive Dyskinesia-like Mouth Movement“, “oral dyskinesia“,"lip mvmts c/w TD, otherwise psychomotor normal", “Tardive dyskinesia: tongue and eyes. “,“TD of the mouth“, “Prominent TD of tongue“, “Tardive dyskinesia versus oral movements due to poor dentition“, “tardive dyskinesia--bucco-oral movements--pt.reports same“, “buccal tardive dyskinesia“, “Some signs of oral tongue movements c/s TD. “,"Mild tardive dyskinesia (orobuccalfacial movements), no rigidity noted, no agitation or retardation", “mild oral TD movements“, “Tardive dyskinesia symptoms present: grinding of teeth and movements of tongue“, “Tardive dyskinesia of tongue more prominent“, “TD (lip smacking) “,“Mild TD movements of mouth/jaw“,“tremulous tongue c/w tardive dyskinesia“,“TD- mouth movements“,“? TD (mouth)“, “normal except for TD of jaw/UL/LL“, “possible oral TD“, “Mouth movements ? TD“, “Orobuccal TD“, “TD in tongue“,“mild TD (orobuccal)“, “Prominent oral lingual movements: chewing, tongue darting, jaw thrusts”, “shakes her left leg up and down rapidly, has oral dyskinesia”, “Slurring in setting of TD”, “tongue dyskinesia”, "TD mostly perioral, tongue + foot tapping", “some TD involving feet”, “TD or hands and legs”, “movement characteristic of TD observed in both hands”, “movements consistent with TD of hands”, “lower extremity TD”, “possible tardive dyskinesia of hands”, “likely tardive dyskinesia—fingers”, "B upper extrem TD, otherwise c", "TD (tongue movement, hand movements and lip smacking)", “Tardive dyskinesia of fingers”, “TD movements in hand and mouth”, “TD in lower extrem”, “TD of upper extremity (variable repetitive flexing of wrist)”, "Tardive dyskinesia, truncal + lower extremities", “finger mvmt suggestive of TD”, “Abnormal movements of feet typical of Tardive Dyskinesia”, "hand tremors, r/o TD", “likely tardive dyskinesia—fingers”, “no eps, +td fingers”, “Hip gyrations (TD-associated?)”, “TD in neck”, “Possible axial TD”, "rocking, ?TD", "Tardive dyskinesia present, rocking," |
| **Abnormal movements which may be associated with tardive dyskinesia** |
| “less pronounced mouth movements“, “mouth movements“, “abnormal oral lingual movements“, , “Mild oral buccal movements“,“noticable oral lingual movements“, “Mouth movements“, “oral movements“, “Slow repetitive movements of jaw“, “Wringing hands”, “Leg jiggling”, “Hand wringing”, “slight hand wringing/restlessness at times”, "some rocking, hand wringing", “rocking”, “Rocking back and forth”, “mild rocking”, “constant truncal rocking”, “mouth movements, rocking in chair”, “Rocking back and forth in chair”, “rocking in her seat”, “rocking movements”, “Rocking movements, restless”, “rocking side to side”, “rocking, oral movements, restless”, “rocks in her chair”, “some rocking”, “Some rocking back and forth in seat”, “Some rocking in chair”, “sporadic rocking in her seat” |

**S Table 4.** All raw text-strings documented within the mental status examination portion of electronic records which may be indicative of tardive dyskinesia (TD)

| **TD symptoms unspecified** |
| --- |
| “Tardive Dyskinesia”, “TD”, “Tardive diskinesia (TD) present”, “evidence of tardive dyskinesia”, “mild TD”, “movements of tardive dyskinesia”, “TD evident”, “TD noted”, “possibly tardive dyskinesia”, “TD like movements that are min. worse from last we”, “Mild Tardive Dyskinesia”, “difficult to read because of tardive, but overall pleasant”, “EPS, and TD”, “marked tardive dyskinesia”, “mild tardive type movements”, “mild TD sx that have greatly improved”, “no abnormal movements aside from mild td-like”, “still tardive dyskinesia”, “td movements”, “TD, missing dentures” |
| **Facial and oral movements** |
| “mild-mod oral-buccal -lingual TD“, “Mild oral dyskinesia“, “Oral dyskinesia“, “less pronounced mouth movements“, “mouth movements“, “abnormal oral lingual movements“, “Tardive Dyskinesia of tongue“, “mouth with tardive syskinesia“, “Mild Tardive Dyskinesia of tongue“, “Mild oral buccal movements“, “Tardive Dyskinesia of mouth“, “noticable oral lingual movements“, “Mouth movements“, “oral movements“, “chronic oral-bucal movments of td“, “TD of mouth“, “Tardive Dyskinesia-like Mouth Movement“, “oral dyskinesia“, "lip mvmts c/w TD, otherwise psychomotor normal", “Slow repetitive movements of jaw“, “Tardive dyskinesia: tongue and eyes. “,“TD of the mouth“, “Prominent TD of tongue“, “Tardive dyskinesia versus oral movements due to poor dentition“, “tardive dyskinesia--bucco-oral movements--pt.reports same“, “buccal tardive dyskinesia“, “Some signs of oral tongue movements c/s TD. “,"Mild tardive dyskinesia (orobuccalfacial movements), no rigidity noted, no agitation or retardation", “mild oral TD movements“, “Tardive dyskinesia symptoms present: grinding of teeth and movements of tongue“, “Tardive dyskinesia of tongue more prominent“, “TD (lip smacking) “,“Mild TD movements of mouth/jaw“, “tremulous tongue c/w tardive dyskinesia“, “TD- mouth movements“, “? TD (mouth)“, “normal except for TD of jaw/UL/LL“, “possible oral TD“, “Mouth movements ? TD“, “Orobuccal TD“, “TD in tongue“, “mild TD (orobuccal)“, “Severe TD orobuccal“, “tardive dyskinesia with orofacial movements“, “tardive dyskinesia with oralfacial movements“, “oral tardive dyskinesia“, “tongue movements consistent with TD“, “Slight tongue darting/Tardive dyskinesia“, "TD upper extremities, oral", “some TD of tongue“, “tardive dyskinesia with chronic involuntary mouth movements“, “?TD in the mouth“, “Facial grimacing/TD present consistent with previous exams.“, "Mild tardive dyskinesia, mostly of tongue", “some 'deepbreaths" suggestive of TD“, “Tardive dyskinesia of the tongue“, “TD of face“, “Possible TD in face; eyebrow movments and occasional lip smacking“, “TD symptoms affecting her mouth. No pillrolling tremor appreciated by me today.“, “orobuccal TD movements“, “questional tardive dyskinesia involuntary movements of lower jaw“, “Oral-buccal TD“, “Improved TD (abnormal lip movements on admission)“ , “restless; possible oral- lingual TD + akathisia“, “mild TD in her lips“, “mild tardive dyskinesia of the mouth“, “R hemifacial tic 2/2 TD“, "nervousness, orolingual movements (TD)", “Fine tremor and mild perioral TD“, “Oral TD“, "mild akasthisia, severe TD orofacial", “perioral movements c/w mild TD“,"TD of face, tongue, BUE", “Tardive dyskinesia (orofacial) “,“TD facial movements improved“, “mild oralbuccal TD“, "Clenched jaw, chattering movement, possible TD", “tardive dyskinesia with lip smacking“, “perioral TD“, “some buccooral dyskinesia suggestive of mild TD“, “involuntary mouth movement consistent with TD“, “Adventitious mouth movements ?TD“,“TD notable esp in jaw“ ,“probable oral TD“, “possible mouth TD“, “mouth/tongue TD“, “some lips smacking noted suggestive of TD“,“TD noted in mouth/tongue/jaw“, “repetitive tongue movements- reports h/o TD that is unchanged“, “possible tardive dyskinesia when speaking“ ,“oral movements: TD“, “ocular and jaw tardive dyskinesias“, “Clicking of mouth (possibly tardive dyskinesia)“, “TD observed in perioral muscles and tongue“, “Perioral tardive dyskinesia“, “Mild tardive dyskinesia“, “minimal oral TD movement“,“? repetitive oral movements; ?TD“, “Mild tardive dyskinesia of tongue“, “TD of tongue“, “Eyelid/Eyebrow twitches most likely TD“,“TD of face/mouth and B/L hand tremor“, "mouth movements, TD", “Oral buccal TD? “,"tardive dyskinesia , perioral movements.", “Oral Buccal TD“, "Tardive dyskinesia, continuous lip smacking", “mild oral-buccal TD movements seen on video“, “dyskinesia of mouth, trunk”, “Prominent oral lingual movements: chewing, tongue darting, jaw thrusts”, “shakes her left leg up and down rapidly, has oral dyskinesia”, “Slurring in setting of TD”, “tongue dyskinesia” |
| **Extremity movements** |
| “Wringing hands”, “Leg jiggling”, “Hand wringing”, “slight hand wringing/restlessness at times”, "some rocking, hand wringing", "TD mostly perioral, tongue + foot tapping", “some TD involving feet”, “TD or hands and legs”, “movement characteristic of TD observed in both hands”, “movements consistent with TD of hands”, “lower extremity TD”, “possible tardive dyskinesia of hands”, “likely tardive dyskinesia—fingers”, "B upper extrem TD, otherwise c", "TD (tongue movement, hand movements and lip smacking)", “Tardive dyskinesia of fingers”, “TD movements in hand and mouth”, “TD in lower extrem”, “TD of upper extremity (variable repetitive flexing of wrist)”, "Tardive dyskinesia, truncal + lower extremities", “finger mvmt suggestive of TD”, “Abnormal movements of feet typical of Tardive Dyskinesia”, "hand tremors, r/o TD", “likely tardive dyskinesia—fingers”, “no eps, +td fingers” |
| **Truncal movements** |
| “rocking”, “Rocking back and forth”, “mild rocking”, “Hip gyrations (TD-associated?)”, “TD in neck”, “Possible axial TD”, "rocking, ?TD", "Tardive dyskinesia present, rocking,", “constant truncal rocking”, “mouth movements, rocking in chair”, “Rocking back and forth in chair”, “rocking in her seat”, “rocking movements”, “Rocking movements, restless”, “rocking side to side”, “rocking, oral movements, restless”, “rocks in her chair”, “some rocking”, “Some rocking back and forth in seat”, “Some rocking in chair”, “sporadic rocking in her seat” |

**S Table 5.** Operational definitions for data extraction

| **Variable** | **Operational definition** |
| --- | --- |
| Autism / Asperger’s Syndrome | ICD-9 code: 299, 299.01  ICD-10 code: F84.0, F84.5 |
| Intellectual disability | ICD-9 code: 317, 318.0, 318.1, 318.2, 319  ICD-10 code: F70, F71, F72, F73, F79 |
| Pervasive and specific developmental disorder | ICD-9 code: 299.1, 299.8, 315, 315.02, 315.1, 315.2, 315.31, 315.32, 315.34, 315.35, 315.39, 315.4, 315.8, 315.9, F80.0, F80.2, F80.81, F80.82, F80.89, F80.9, ICD-10 code: F81.0, F81.2, F81.81, F82, F84.8, F84.9, F88, F89 |
| Obsessive compulsive disorder | ICD-9 code: 300.3, 300.30  ICD-10 code: F42.0, F42.1, F42.2, F42.8, F42.9 |
| Anticholinergic prescription* | Benztropine, benzhexol, biperiden, orphenadrine, procyclidine, scopolamine, trihexyphenidyl |
| First generation antipsychotic | Chlorpromazine, droperidol, fluphenazine,  haloperidol, loxapine, mesoridazine, molindone,  perphenazine, pimozide, prochlorperazine, thioridazine, thiothixene, trifluoperazine, zuclopenthixol, benperidol, chlorprothixene, perazine, sulpiride |
| Second generation antipsychotic | Aripiprazole, asenapine, brexpiprazole, cariprazine, iloperidone, lumateperone, lurasidone, olanzapine, paliperidone, quetiapine, risperidone, ziprasidone, clozapine, amisulpride, quetiapine |

*Anticholinergic drugs used to treat movement disorders like parkinsonism and dystonia sourced from the review by Bergman and colleagues (Bergman & Soares‐Weiser, 2018).

**S Table 6.** Output from logistic regression analyses to investigate associations between demographic and clinical characteristics and presence of TD ICD diagnosis in the sub-cohort of patients with presence of TD recorded in semi-structured portions of the EHR (n = 610)

| **Variable** | **N (%)** | **Univariate** | | **Multivariable** | |
| --- | --- | --- | --- | --- | --- |
|  |  | **OR (95% CI)** | **p-value** | **OR (95% CI)** | **p-value** |
| Age (mean ± SD) | 51.4 ± 12.7 | 1.01 (0.99-1.03) | 0.46 | 1.01 (0.98-1.03) | 0.59 |
| Female sex | 345 (56.6) | 0.88 (0.50-1.53) | 0.63 | 0.80 (0.45-1.43) | 0.45 |
| Race   - White (reference) - Black - Asian - Other - Unknown | 302 (49.5)  173 (28.4)  4 (0.7)  18 (3.0)  113 (18,5) | -  0.39 (0.17-0.80)  2.39 (0.12-19.2)  0.90 (0.14-3.32)  0.47 (0.19-1.03) | -  0.02  0.46  0.89  0.08 | -  0.45 (0.19-0.97)  1.70 (0.08-16.6)  1.00 (0.15-3.96)  0.54 (0.20-1.26) | -  0.05  0.67  1.00  0.17 |
| First-generation antipsychotic | 205 (33.6) | 1.01 (0.56-1.79) | 0.96 | 1.35 (0.30-9.12) | 0.69 |
| Second-generation antipsychotic | 478 (78.4) | 0.73 (0.40-1.41) | 0.33 | 1.23 (0.25-6.24) | 0.80 |
| Multiple antipsychotics | 143 (23.4) | 0.44 (0.18-0.93) | 0.05 | 0.35 (0.10-0.98) | 0.07 |
| Anticholinergic | 181 (29.7) | 1.14 (0.62-2.02) | 0.67 | 1.16 (0.61-2.17) | 0.64 |
| Clinical setting   - AMC - CMHC - PP | 397 (65.1)  126 (20.7)  87 (14.3) | -  2.94 (1.62-5.32)  0.80 (0.27-1.98) | -  <0.001  0.66 | -  2.39 (1.24-4.61)  0.62 (0.20-1.66) | -  0.01  0.38 |

Outcome = ICD-TD code present (yes, n = 56; no, n = 554). Reference categories for multilevel categorical variables were selected as the category with the highest number of observations. MSE = mental state examination, AMC = academic medical centre, CMHC = community mental health centre, PP = private practice. Other race = American-Indian or Alaska Native, Native Hawaiian or Other Pacific Islander, multiracial. No individuals treated at a substance use disorder treatment centre were included in the sub-cohort..

**SUPPLEMENT REFERENCES**

Bergman, H., Walker, D.-M., Nikolakopoulou, A., Soares-Weiser, K., & Adams, C. E. (2017). Systematic review of interventions for treating or preventing antipsychotic-induced tardive dyskinesia. *Health technology assessment*, *21*(43), 1-218.
